# Determining the optimal cut-off scores for the Chinese version of the Memorial Anxiety Scale for Prostate Cancer (MAX-PC)

**DOI:** 10.1186/s12955-023-02210-1

**Published:** 2023-12-01

**Authors:** Qingmei Huang, Ping Jiang, Yuanqi Ding, Yaning Zheng, Li Zheng, Jie Luo, Yun Dai, Fulei Wu, Wei Wang

**Affiliations:** 1 School of Nursing, Fudan University, Shanghai, China; 2 The International Peace Maternity and Child Health Hospital, School of Medicine, Shanghai Jiao Tong University, Shanghai, China; 3 Department of Nursing, Nanjing Drum Tower hospital, Nanjing, China; 4 Department of Nursing, First Affiliated Hospital, School of Medicine, Zhejiang University, Zhejiang, Hangzhou China; 5 Department of Urology, First Affiliated Hospital, School of Medicine, Zhejiang University, Zhejiang, Hangzhou China

**Keywords:** Prostate cancer, Memorial anxiety scale, Cut-off score, Receiver operating characteristic curve

## Abstract

**Purpose:**

Anxiety is a common emotion experienced by patients with prostate cancer (PCa), and can be exacerbated by testing the prostate-specific antigen (PSA) index. The Memorial Anxiety Scale for Prostate Cancer (MAX-PC) was developed to assess the cancer-specific anxiety of these patients, but lack of appropriate thresholds for this scale limits its use. This study aimed to utilize ROC curve analysis to identify the best cut-off values for the Chinese version of the MAX-PC scale.

**Methods:**

A cross-sectional survey was conducted using the Chinese version of the MAX-PC scale and the Generalized Anxiety Disorder Scale (GAD). ROC curve analysis, Youden index, Kappa consistency test and McNemar test were used for the optimal cutoff points for screening mild, moderate, and severe cancer-specific anxiety levels in patients with PCa, on the Chinese version of the MAX-PC scale.

**Results:**

Two hundred eighty-seven patients with PCa completed the survey. The appropriate cut-off values for the MAX-PC scale for screening patients with PCa for cancer-specific anxiety were 20, 28, and 38 for mild, moderate, and severe anxiety, respectively with the highest Youden indices. The Kappa and McNemar’s test showed the best level of consistency with values of 0.627, 0.580, and 0.606 for screening mild, moderate, and severe anxiety, respectively.

**Conclusions:**

The scores 20, 28, and 38 are the best cut-off values for the Chinese version of the MAX-PC scale. This scale should be used for screening cancer-specific anxiety for patients with PCa to assess and evaluate their anxiety levels and provide targeted interventions.

## Introduction

Prostate cancer (PCa) has been reported to be the most prevalent cancer in men worldwide [1]. Diagnosis and treatment of PCa cause not only physical complications, such as urinary incontinence, urinary tract irritation, and erectile dysfunction [2], but also mental health issues. Anxiety is the most frequently reported negative emotion among patients with PCa, with incidence rates ranging from 15.09 to 32.6%, as revealed by various studies employing different anxiety assessment tools [3, 4]. In patients with PCa, regular testing of the prostate-specific antigen (PSA) index is essential to monitor treatment outcomes and disease progression [5]. Anxiety is a common emotion experienced by these individuals, which can be exacerbated by elevated PSA levels. Furthermore, Roth et al. discovered that many patients with PCa are reluctant to discuss their feelings, and instead, tend to ask excessive questions about their treatment or prognosis, or delay or repeat PSA tests to express their anxiety [6]. This particular type of anxiety was identified as prostate cancer-specific anxiety, which were unique to men with PCa and were specifically centered on their illness itself or PSA testing [6–8].

To better identify and measure anxiety states associated with PCa, Roth developed the Memorial Anxiety Scale for Prostate Cancer (MAX-PC) in 2003 [6]. This 18-item scale assesses cancer-specific anxiety in three dimensions: prostate cancer anxiety, anxiety related to PSA testing, and fear of cancer recurrence. It reportedly has an internal consistency reliability of 0.89 and a retest reliability of 0.89 [6, 7]. However, the scale does not provide a criterion for assessing the magnitude of anxiety scores of patients with PCa, that is, it is difficult to determine which patients attain clinically meaningful scores and need further clinical attention. The MAX-PC scale was revised again in 2006 and in the results, the researchers directly applied a cutoff of 27 to identify PCa Patients with clinically significant anxiety, which represents an average rating of 1.5 on the 0–3 rating scale [9]. Unfortunately, this empirically derived approach to determining the appropriate scale threshold lacks support from objective data, which has led subsequent researchers to report different threshold values when using the scale. For example, in the study by Tavlarides et al., a score of MAX-PC ≥27 indicated a high level of anxiety, a score of MAX-PC < 4 indicated a low level of anxiety [10]. However, study by Tan Hung-Jui et al., a MAX-PC score of ≥26 was used as a cut-off point [11].

Appropriate cutoff values are essential for the optimization of the scale’s screening accuracy and enhancement of the scale’s sensitivity to what is being measured [12, 13]. A low threshold may lead to a high rate of misdiagnosis, misdiagnosing healthy people as having anxiety disorders, whereas a high threshold may result in more false negatives, thus missing diagnoses of those with anxiety issues. Yet now, the use of aforementioned cutoff values has not been validated by rigorous data. Therefore, it is crucial to utilize scientific and objective research methods to determine the appropriate threshold values for the scale to establish screening criteria for the scale, as well as to improve the value of the scale and lay the groundwork for its wider and more practical use in both clinical and research settings.

Receiver operating characteristic (ROC) curve analysis is a widely used statistical method for establishing the optimal threshold of a scale. This technique can be used to measure the sensitivity and specificity of different diagnostic cutoff points on the curve, where each point corresponds to the corresponding sensitivity and specificity. When the sensitivity and specificity are high, the corresponding point is considered the optimal demarcation point of the scale. The area under the ROC curve can be used to assess the accuracy of a diagnostic test [14, 15]. In ROC diagnostic test design, a gold standard is needed for reference, and the Generalized Anxiety Disorder Scale (GAD) scale serves this purpose. Developed from the seven diagnostic criteria of the Diagnmental and Statistical Manual of Mental Disorders Fourth Edition (DSM-IV) [16], GAD-7 is an effective and concise tool for assessing and screening for anxiety disorder. It is widely used in clinical practice, and has established cut-off values [17]. Therefore, this study aimed to utilize ROC curve analysis with GAD-7 as “gold standard” to identify appropriate cut-off value for the Chinese version of the MAX-PC scale, thereby establishing criteria for the scale to screen for cancer-specific anxiety levels in patients with PCa.

## Methods

### Study design and participants

This was a cross-sectional study with data collected from November 2016 to January 2017 at the Department of Urology of a tertiary care hospital in Zhejiang Province using convenience sampling. The study population included patients who met the following criteria: 1) PCa diagnosis through prostate puncture biopsy or surgical pathology results, 2) ability to communicate effectively in Mandarin, and 3) willingness to participate in the study after being informed about it and providing consent. Patients were excluded if they met any of the following criteria: 1) unknown condition, 2) concurrent presence of other types of tumors, 3) concurrent presence of other serious complications, and 4) combined psychiatric disorders. This study was approved by the Ethics Committee of the First Affiliated Hospital of Zhejiang University School of Medicine, and all participants provided informed consent.

### Data collection

Investigators in this study conducted training to standardize how they explained and reviewed the questionnaire items. After obtaining informed consent from eligible patients, the participants were given a packet of self-reported questionnaires on paper to complete. Questionnaires were collected and sent back on-site after quality review. For those with poor literacy or eyesight, two trained investigators conducted face to face interviews.

### Instruments

#### Demographic and clinical characteristics

A self-designed questionnaire was used to collect demographic and clinical information, including age, marital status, occupational status, location of residence, education, recent PSA level, PCa family history, and treatment method.

#### Chinese version of MAX-PC

The MAX-PC was developed by Roth et al. to identify and assess cancer-specific anxiety in men with PCa [6]. It comprises 18 items divided into three subscales: general PCa anxiety, anxiety related to PSA testing (PSA anxiety), and fear of recurrence. The scores range from 0 to 54 on the total scale, with higher scores indicating higher levels of anxiety [7, 9]. The Chinese version of MAX-PC had been translated by our team who had evaluated its psychometric properties in Chinese men, with Cronbach’s alpha coefficient for the total and the three subscales being 0.94, 0.93, 0.82, and 0.85, respectively [8].

#### Chinese version of GAD-7

The GAD-7 scale was originally developed by Spitzer et al. in 2006 and comprises seven items based on the seven diagnostic criteria of the DSM-IV [16]. The scale measures a single dimension with scores ranging from 0 to 3 for each item and 0–21 for the total score. The GAD-7 is a clinical tool used to screen for anxiety disorders and monitor treatment outcomes. The scale employs a scoring system of 0–4 indicating no anxiety, 5–9 indicating mild anxiety, 10–14 indicating moderate anxiety, and 15–21 indicating severe anxiety. The Cronbach’s alpha coefficient for Chinese version of GAD-7 was 0.859 [18]. In this study, the aforementioned scoring criteria were utilized, with cut-off values of 5, 10, and 15 for mild, moderate, and severe anxiety, respectively [17].

### Statistical analysis

ROC curve analysis was used to test the ability of the MAX-PC to discriminate between patients with and without PCa-related anxiety. The following indicators were calculated: area under the curve (AUC), sensitivity, specificity, total consistency rate, diagnostic error rate, and diagnostic omission rate. The Youden index was also calculated in conjunction with the above results to select the optimal cutoff values for MAX-PC. The optimal cutoff values, located at the top-left point of the ROC curve, were derived in each curve from the point with the maximum Youden index, which represented the maximized sensitivity and specificity [14, 15]. Additionally, the Kappa consistency test was used to compare the degree of agreement between the two evaluation tools for the diagnosis of anxiety status in patients with PCa. The larger the Kappa score, the better the consistency. The specific evaluation criteria are as follows: Kappa ≤0.2, indicating poor consistency; 0.2 < Kappa ≤0.4, indicating average consistency; 0.4 < Kappa ≤0.6, indicating medium consistency level; 0.6 < Kappa ≤0.8, indicating good consistency level; Kappa > 0.8, indicating very good consistency [19]. In addition, the McNemar test, which is a chi-square test designed for paired count data, was employed to determine whether there was a statistically significant difference between the outcomes of the two assessment instruments, MAX-PC and GAD-7, in detecting anxiety among patients with PCa. All tests were two-tailed, and a *p* value < 0.05 was considered statistically significant. Statistical analyses were performed using the SPSS software (version 17.0).

## Results

### Participants’ characteristics

During the study period, 287 patients with PCa consented to participate and completed the study. Their mean age was 68.41 ± 7.97 years (range 30 ~ 88 years), and 181 patients (63.1%) were over 65 years; 237 patients (82.6%) were retirees; degree of education, 104 patients (36.2%) were college level or above; 200 patients (69.7%) were from urban areas. The PSA detection indicators above 20.000 ng/ml accounted for 11.1% of the cases. Table 1 summarizes the main sociodemographic and clinical characteristics of the participants.

**Table 1:** Sociodemographic and clinical characteristics of the sample

Variables	N	%
**Age (years)**		
< 60	31	10.8
60–65	75	26.1
66–70	70	24.4
> 70	111	38.7
**Marital status**		
Married	275	95.8
Divorced, single, widowed	12	4.2
**Occupational status**		
Employed	50	17.4
Retired	237	82.6
**Residence**		
Village or countryside	30	10.5
County	57	19.9
City	200	69.7
**Education**		
Primary school or below	43	15.0
Junior high school	70	24.4
Senior high school	70	24.4
College graduate or above	104	36.2
**Recent PSA (ng/ml)**		
≤ 4.000	147	51.2
4.001–10.000	58	20.2
10.001–20.000	44	15.3
> 20.000	32	11.1
Missing	6	2.1
**Family history of PCa**		
Yes	14	4.9
No	273	95.1
**Treatment method**^a^		
Open radical prostatectomy	60	20.9
Laparoscopic radical prostatectomy	35	12.2
Robotic-assisted radical prostatectomy	107	37.3
Particle implantation	13	4.5
Endocrine therapy	89	31.0
Radiation therapy	82	28.6
Orchiectomy	11	3.8
Chemotherapy	11	3.8

^a^multiple-choice question

### Cut-off value for screening mild anxiety of MAX-PC scale

**Fig. 1:**
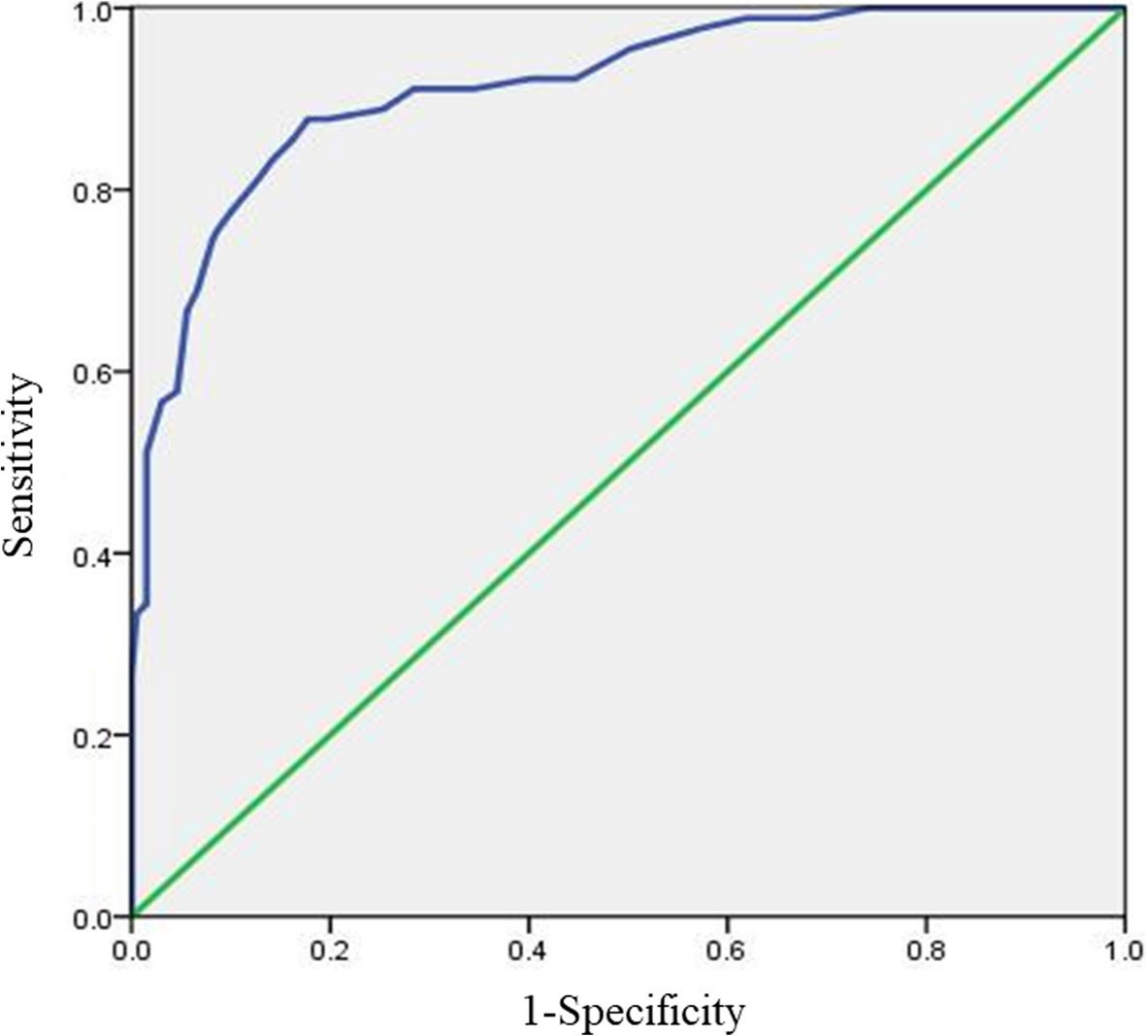
ROC curve for mild anxiety cut-off value of MAX-PC

The cutoff values for diagnosing mild, moderate, and severe anxiety on the GAD-7 scale were 5, 10, and 15 points, respectively, which were used as criteria for the ROC curve analysis to determine the optimal cutoff points for screening mild, moderate, and severe cancer-specific anxiety levels in patients with PCa on the Chinese version of the MAX-PC scale. First, the ROC curve was generated with a total GAD − 7 score of 5 as the threshold, and the results showed that the MAX-PC had a value of 20 with the maximum Youden index, which corresponded to a sensitivity of 87.8%, a specificity of 82.2%, a misdiagnosis rate of 17.8%, and an omission diagnostic rate of 12.2%. Therefore, 20 was determined to be the optimal cutoff value for the MAX-PC scale to assess patients with PCa in a mild anxiety state. See Table 2 and Fig. 1.

**Table 2:** Cut-off value for mild anxiety of MAX-PC

Cut-off value	Sensi-tivity	Specificity	Mistake diagnosis rate	Omission diagnosis rate	Youden Index
18	88.9%	74.6%	25.4%	11.1%	0.635
19	87.8%	80.2%	19.8%	12.2%	0.680
20	87.8%	82.2%	17.8%	12.2%	0.700
21	85.6%	83.8%	16.2%	14.4%	0.694
22	83.3%	85.8%	14.2%	16.7%	0.691
23	81.1%	87.3%	12.7%	18.9%	0.684

### Cut-off value for screening moderate anxiety of MAX-PC scale

**Fig. 2:**
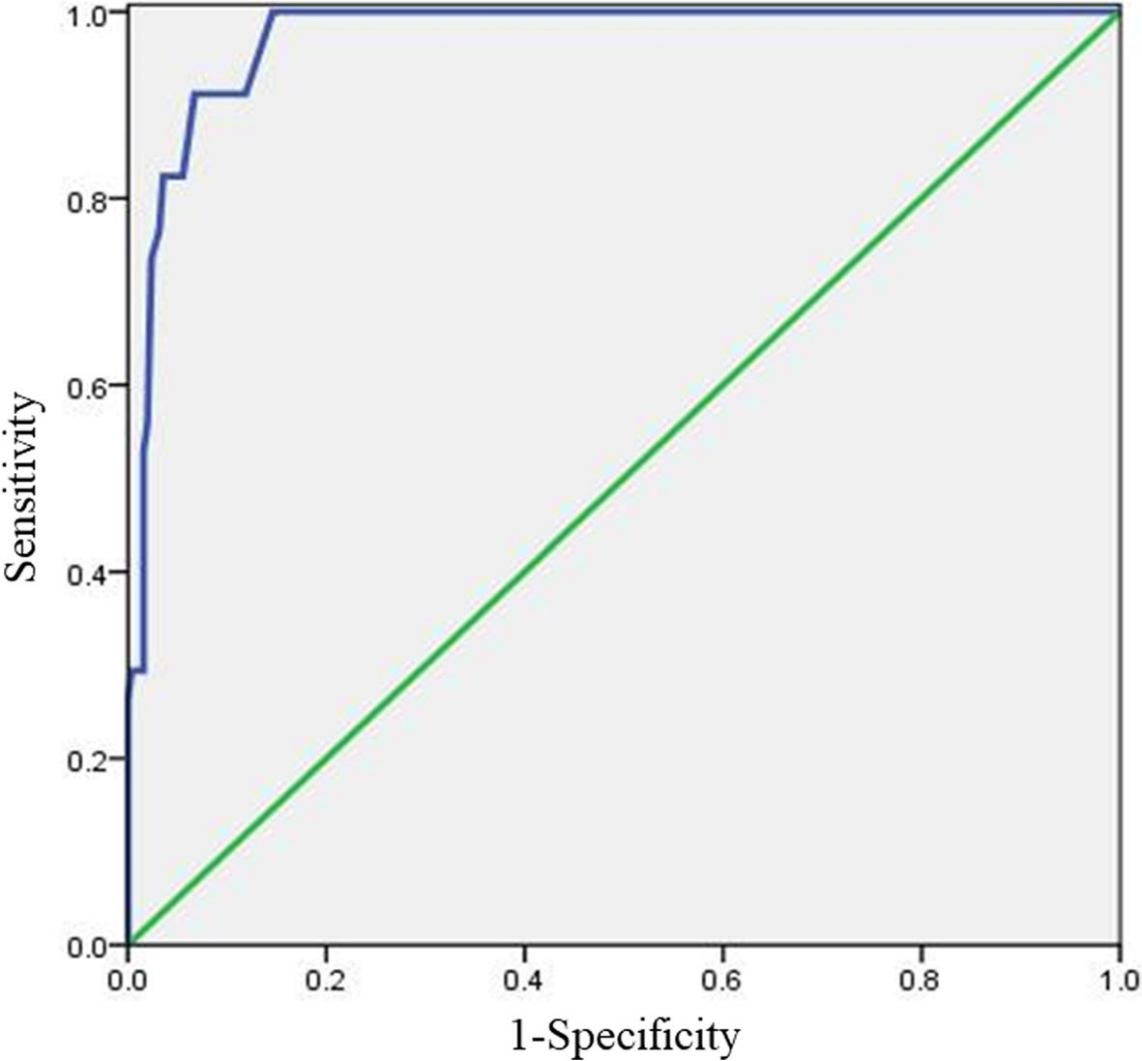
ROC curve for moderate anxiety cut-off value of MAX-PC

A total GAD-7 score of 10 was set as the threshold. ROC analysis revealed that a MAX-PC score of 28 points had the highest Youden index. This score had a sensitivity of 100.0%, specificity of 85.4%, misdiagnosis rate of 14.6%, and missed diagnosis rate of 0.0%. Thus, a threshold value of 28 points was identified as the optimal cutoff point for identifying patients with PCa with moderate anxiety using the MAX-PC scale. See Table 3 and Fig. 2.

**Table 3:** Cut-off value for moderate anxiety of MAX-PC

Cut-off value	Sensitivity	specificity	Mistake diagnosis rate	Omission diagnosis rate	Youden Index
25	100.0%	79.8%	20.2%	0.0%	0.798
26	100.0%	80.6%	19.4%	0.0%	0.806
27	100.0%	83.8%	16.2%	0.0%	0.838
28	100.0%	85.4%	14.6%	0.0%	0.854
29	91.2%	88.1%	11.9%	8.8%	0.793
30	91.2%	89.7%	10.3%	8.8%	0.809
31	91.2%	92.9%	7.1%	8.8%	0.841

### Cut-off value for screening severe anxiety of MAX-PC scale

**Fig. 3:**
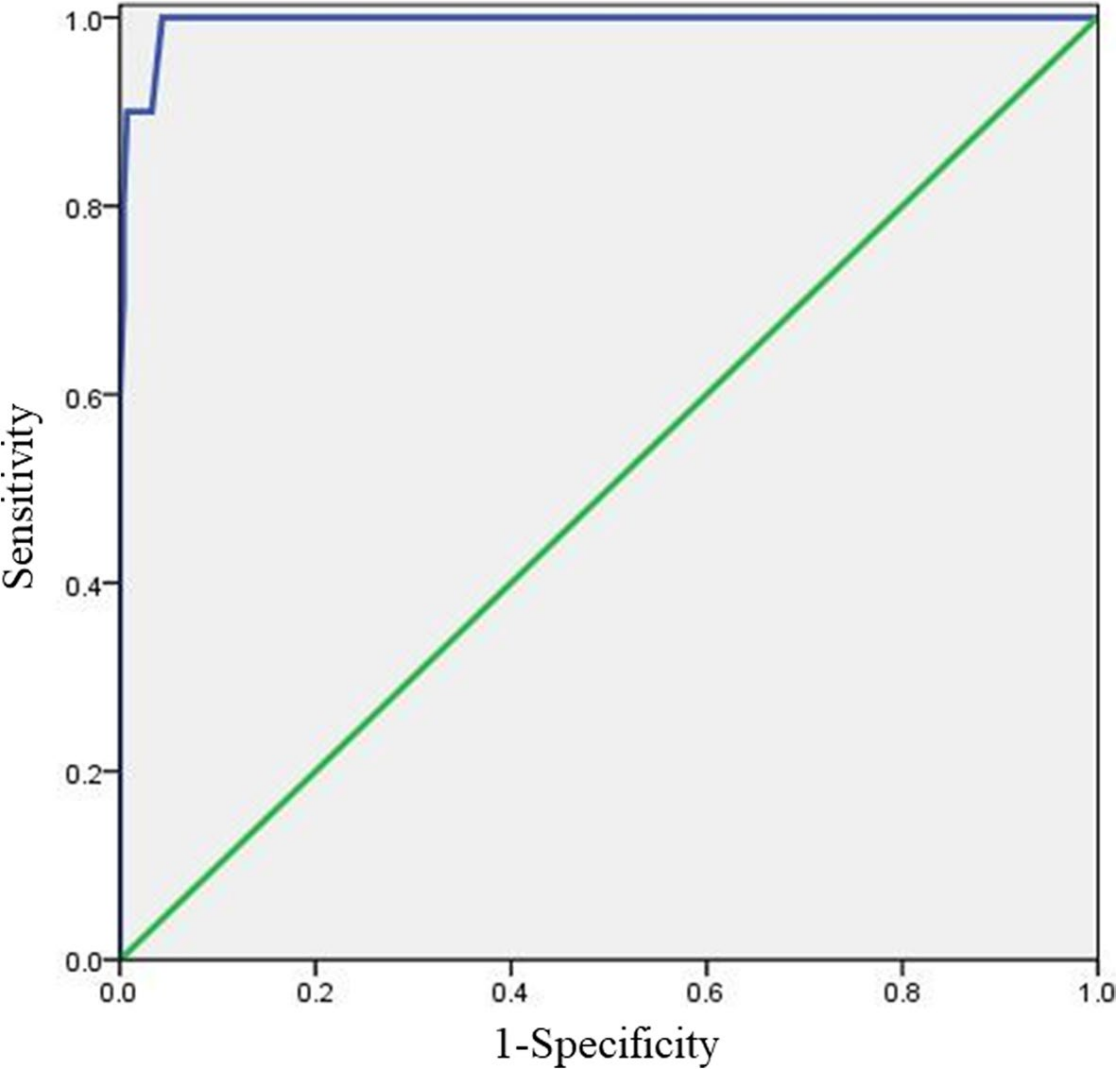
ROC curve for severe anxiety cut-off value of MAX-PC

Finally, a total GAD-7 score of 15 was used as the threshold. In the ROC analysis, a MAX-PC score of 38 points was found to have the highest Youden index. This score had a sensitivity of 100.0%, specificity of 95.7%, misdiagnosis rate of 4.3%, and missed diagnosis rate of 0.0%. Consequently, a threshold value of 38 points was deemed the optimal cutoff point for identifying patients with PCa and severe anxiety using the MAX-PC scale. See Table 4 and Fig. 3.

**Table 4:** cut-off value for severe anxiety of MAX-PC

Cut-off value	Sensi-tivity	Specificity	Mistake diagnosis rate	Omission diagnosis rate	Youden Index
35	100.0%	91.3%	8.7%	0.0%	0.913
36	100.0%	92.4%	7.6%	0.0%	0.924
37	100.0%	94.9%	5.1%	0.0%	0.949
38	100.0%	95.7%	4.3%	0.0%	0.957
39	90.0%	96.8%	3.2%	10.0%	0.868
40	90.0%	96.8%	3.2%	10.0%	0.868
41	90.0%	97.1%	2.9%	10.0%	0.871

Out of the 287 participants who took part in this study, 43 (15.0%) showed mild anxiety (score ranging from 20 to 27), 49 (17.1%) had moderate anxiety (score ranging from 28 to 37), and 22 (7.7%) had severe anxiety (score ≥ 38), according to the cut-off values of MAX-PC.

### Kappa consistency test and McNemar test results

The Kappa consistency test and McNemar’s test were used to evaluate the consistency level of screening anxiety in patients with PCa using the MAX-PC and GAD-7 scales, respectively. According to the results, the two instruments had the best level of consistency in screening mild anxiety, with a Kappa value of 0.627. The level of consistency in screening moderate anxiety was moderate, not reaching above 0.6, and the consistency in screening severe anxiety was also appropriate (0.606). However, the McNemar test results for all three screening methods were significantly different, indicating that, although the consistency of the screening results of the two screening instruments was satisfactory, there were still differences. See Table 5.

**Table 5:** The degree of consistency in screening PCa patients for anxiety across thresholds of the MAX-PC scale (compared with GAD-7 diagnostic results)

MAX-PC scale cut-off value	Consistency test		McNemar test *P*
	Kappa value	*P*	
Mild-20	0.627	< 0.001	< 0.001
Moderate-28	0.580	< 0.001	< 0.001
Severe-38	0.606	< 0.001	< 0.001

## Discussion

Appropriate threshold values are an important prerequisite for maximizing the value of the scale use, effectiveness of scale screening, and sensitivity of the scale to what is being measured [12, 13]. Although Roth et al. developed the MAX-PC scale and validated its reliability, the researchers did not conduct a rigorous diagnostic test design and evaluation to establish appropriate threshold values for the scale [6, 7, 9]. This study used the GAD-7 scale as the gold standard and determined the optimal cut-off values for the MAX-PC scale for mild, moderate, and severe anxiety by ROC curve analysis. This is the first study to evaluate and analyze the appropriate cut-off values for the MAX-PC scale in a diagnostic test, which has important implications for the use of this scale to screen the mental health of PCa patients.

The results of this study showed that the appropriate cut-off values for the MAX-PC scale for screening PCa patients for cancer-specific anxiety were 20 for mild anxiety, 28 for moderate anxiety, and 38 for severe anxiety. The diagnostic sensitivity and specificity of all three cutoff values were high, and the area under the ROC curve was above 0.9, indicating that all of them had high screening accuracy. Compared to previous reports, for example, in study of Roth et al., the appropriate cut-off of the MAX-PC total scale was set at 27 points [9], and in study by Tan Hung-Jui et al., a MAX-PC score of ≥26 was used as a cut-off point to classify the presence of anxiety disorders in patients [11], they both used a single cut-off value as the only criterion for evaluating cancer-specific anxiety in men with PCa. However, the single-dimensional cut-off value is too high for mild anxiety and too low for severe anxiety, making it difficult to differentiate between individuals with varying levels of anxiety and determine the best course of action for each patient. Nevertheless, study by Tavlarides et al. used multi-dimensional cut-off values, in contrast to the results of our study, they used cut-off of MAX-PC ≥27 as high level of anxiety, < 4 as a low level of anxiety, and a score 4–27 as moderate level of anxiety [10]. Setting the scoring standard too low may prevent the overlooking of patients, however it could also result in an overestimation of patients’ anxiety, leading to the misallocation of medical resources. In addition, it is noteworthy to point out that the cut-off values determined in these past studies have not been verified by a rigorous diagnostic test design and lack the support of rigorous scientific data. The robust diagnostic test design and analysis used in this study, providing rigorous and objective evaluation data for the establishment of appropriate cutoff values for the MAX-PC scale, is its main strength. Compared with the empirically derived cutoff score, the results of this study will promote the further application of the MAX-PC scale. However, there are differences in the optimal cutoff values for the same scale in different cultural background groups [20, 21]; therefore, we hope that more researchers will construct appropriate cutoff values for other language versions of the MAX-PC scale to further validate or compare the results of this study.

Overall, in this study, we determined the cutoff values for mild, moderate, and severe anxiety in patients screened using the MAX-PC scale, which can help more accurately assess and evaluate the anxiety levels of PCa patients and provide targeted interventions. In the future, patients with MAX-PC scores < 20 can be considered to have no anxiety, and thus, may not need psychological support, whereas patients with MAX-PC scores between 20 and 27 may indicate that they have mild anxiety and can be screened early and given psychological support. Patients with moderate anxiety with a score between 28 and 37 should be given focused attention and psychological support for them to gradually improve to a mild or anxiety-free state. For patients with PCa with a score of 38 or above, continuous attention and psychosocial support should be provided to prevent patients from developing chronic anxiety disorders and affecting the long-term quality of life of survivors.

This study has a few limitations. First, the participants in our study were mainly recruited from the Zhejiang Province of China for convenience sampling, and 69.7% of the patients were from urban areas. Imparity in the regions of origin of the study participants introduces a degree of selection bias in the inclusion of participants, thereby constraining the representativeness of our results to other geographic regions. Furthermore, while the established cutoff values for mild, moderate, and severe anxiety on the MAX-PC scale demonstrated high screening sensitivity and specificity, and the corresponding area under the ROC curve indicated high screening accuracy, the Kappa values obtained from the Kappa consistency test were not particularly high. Additionally, McNemar’s test results revealed significant differences, suggesting that the screening outcomes of the two instruments were dissimilar. Consequently, further research is necessary to investigate the appropriate threshold values for the Chinese version of the MAX-PC scale to enhance the consistency of the diagnostic results. Finally, the MAX-PC scale was primarily utilized as a preliminary screening tool to evaluate the patients’ anxiety status. Its classification of mild, moderate, and severe anxiety levels is intended to identify high-risk groups and facilitate early intervention to prevent further deterioration. However, notably, screening results cannot be used for a definitive diagnosis. A professional mental health doctor must conduct a structured clinical interview using DSM-IV as the standard to confirm the diagnosis of an anxiety disorder.

## Conclusion

To summarize, this investigation marks the first instance of creating and evaluating a meticulous diagnostic examination that established effective cutoff points for detecting mild, moderate, and severe cancer-related anxiety using the MAX-PC scale. This study provided objective and robust evidence to support the development of the critical value for the MAX-PC scale and established the parameters for identifying varying levels of anxiety in patients with PCa using this scale. The usefulness of the scale has been enhanced, paving the way for its extensive use in both clinical and scientific research.

## Data Availability

The datasets generated and/or analyzed in the current study are available from the corresponding author upon reasonable request.
